# Spontaneous Regression of a Cholesterol Granuloma in a Mushroom-Like Extension Into the Middle Cranial Fossa After Tympanoplasty

**DOI:** 10.7759/cureus.111865

**Published:** 2026-07-01

**Authors:** Satoshi Tsuruta, Masaharu Sakagami, Tadashi Nishimura, Tadashi Kitahara

**Affiliations:** 1 Otolaryngology - Head and Neck Surgery, Nara Medical University, Kashihara, JPN

**Keywords:** cholesteatoma, cholesterol granuloma, intracranial lesion, middle ear, tympanoplasty

## Abstract

Intracranial extension of a middle ear cholesterol granuloma is rare and typically requires invasive neurosurgical resection. This case is unique as it demonstrates spontaneous regression of a middle cranial fossa lesion following a minimally invasive transmastoid approach. A 41-year-old man presented with a suspected recurrent cholesteatoma. Magnetic resonance imaging (MRI) revealed a "mushroom-like" lesion extending into the middle cranial fossa, showing high signal intensity on both T1- and T2-weighted images without diffusion restriction, diagnostic of cholesterol granuloma. To minimize risk in the dominant hemisphere, we performed a transmastoid removal of the middle ear lesion and closed the bony defect with bone paté, leaving the intracranial portion. Postoperative MRI at four months confirmed complete disappearance of the intracranial lesion. Managing the middle ear environment and blocking the communication route could trigger spontaneous regression of intracranial cholesterol granulomas, providing a viable, less invasive alternative to direct intracranial surgery.

## Introduction

Cholesterol granuloma is a foreign body reaction to cholesterol crystals derived from the breakdown products of retained exudate or blood components and is thought to arise in association with impaired ventilation or drainage obstruction in pneumatized bony spaces, particularly the temporal bone [1-3]. Clinically, middle ear lesions are often identified incidentally during surgery for chronic otitis media or cholesteatoma, although some lesions may show locally aggressive behavior [1,2]. In rare instances, aggressive temporal bone cholesterol granulomas may extend intracranially [4-10]. In such cases, surgical management has generally been regarded as the standard treatment, with most previously reported cases undergoing surgical resection [5-10]. We herein report a rare case of middle ear cholesterol granuloma with a small, mushroom-like extension into the middle cranial fossa that spontaneously regressed after tympanoplasty.

## Case presentation

The patient was a 41-year-old man who had previously undergone type I tympanoplasty for left cholesteatoma at another hospital. Follow-up showed no recurrence for two years, but the patient had temporarily suspended outpatient visits due to the COVID-19 pandemic. Subsequently, during magnetic resonance imaging (MRI) performed at another hospital for follow-up of an internal carotid artery aneurysm, an incidental finding revealed a lesion enlarging from the mastoid cavity into the intracranial space over six months. Suspecting recurrence of cholesteatoma, the patient was referred to our hospital for consultation five years after the initial surgery.

Imaging findings

MRI revealed high signal intensity on both T1-weighted (Figure 1A) and T2-weighted (Figure 1B) images. Diffusion-weighted imaging (DWI) showed no restricted diffusion. Based on these findings, the diagnosis was revised from recurrent cholesteatoma to intracranial extension of a cholesterol granuloma. CT revealed soft-tissue lesions in the attic and mastoid, accompanied by a partial bony defect in the middle cranial fossa (Figures 2A, 2B).

**Figure 1:**
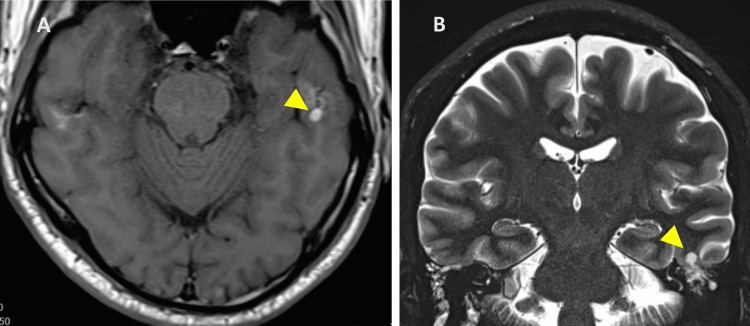
Preoperative MRI images (A) T1-weighted image. A spherical area of high signal intensity was observed intracranially (yellow arrowhead). (B) T2-weighted image. A "mushroom-like" hyperintense area extending intracranially was observed (yellow arrowhead).

**Figure 2:**
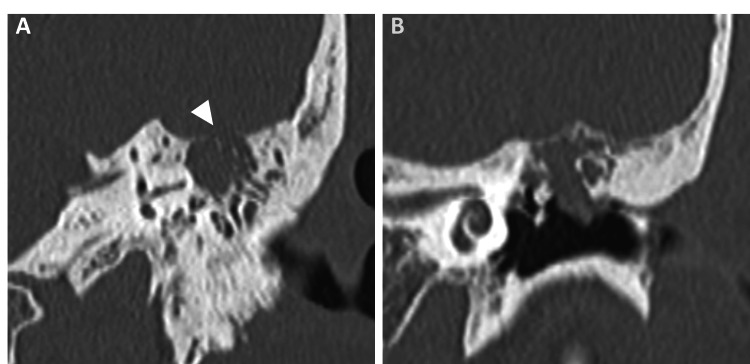
Preoperative CT images Partial bony defect of the middle cranial fossa (A, white arrowhead) and soft tissue lesions in the attic and mastoid (A, B).

Treatment strategy

After consultation with the neurosurgery department, it was determined that surgical resection posed a high risk because the intracranial lesion was small but located in the dominant hemisphere. Therefore, the surgical strategy focused on removing the middle ear lesion while monitoring the intracranial portion. Removal of the intracranial lesion would be considered only in the event of marked enlargement, surrounding edema, or the onset of neurological symptoms. The surgery was planned via a transmastoid approach, prioritizing the preservation of intracranial structures.

Surgical findings

Inspection of the middle ear cavity via the transmastoid approach revealed no recurrence of cholesteatoma; only granulomatous tissue was observed (Figure 3A). The granuloma and glistening soft tissue were observed in the middle ear cavity (Figure 3B). During removal of the glistening soft tissue, endoscopic observation revealed soft tissue pulsating in synchrony with the dura mater, and a bone defect in the middle cranial fossa was identified (Figure 3C). The soft tissue was removed as much as possible while avoiding dural injury. To block communication between the middle ear and the intracranial space, the bone defect was sealed with bone paté, isolating the remaining soft tissue (Figure 3D).

**Figure 3:**
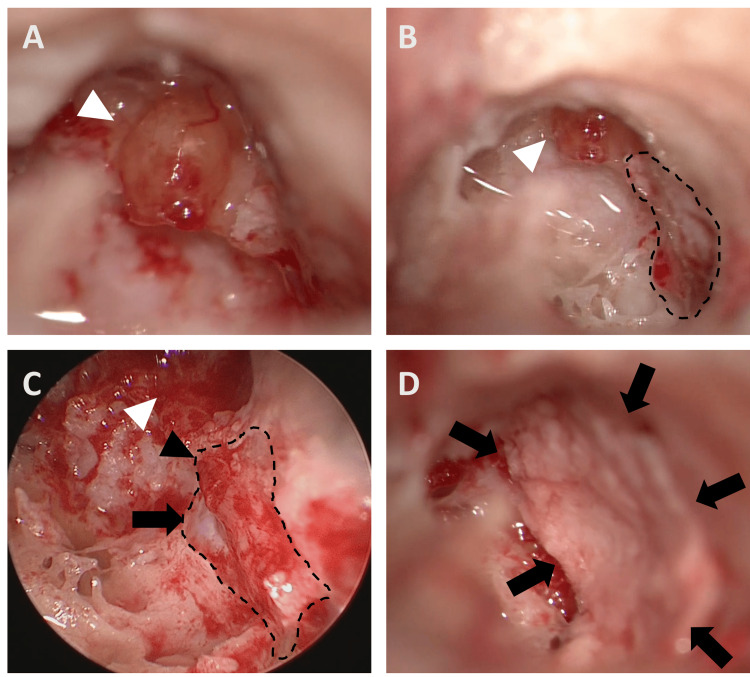
Intraoperative findings (A) View of the middle ear cavity via the transmastoid approach. No recurrence of cholesteatoma was seen in the middle ear cavity; only granuloma (white arrowhead) was confirmed. (B) View of the middle ear cavity via the transmastoid approach. The granuloma (white arrowhead) and glistening soft tissue (dotted line) were observed; the glistening soft tissue was confirmed as a cholesterol granuloma on histopathology. (C) Endoscopic findings. Behind the area where the granuloma was removed (white arrowhead), a bone defect of the middle cranial fossa (dotted line) was identified. Soft tissue suspected to be the lesion (black arrowhead) and partial exposure of the dura mater (black arrow) were observed within the defect. The soft tissue pulsated in synchrony with the dura, making total resection difficult. (D) View of the middle ear cavity via the transmastoid approach. The bone defect of the middle cranial fossa was closed using bone paté (black arrows) to block communication between the middle ear cavity and the intracranial space.

Histopathological examination revealed cholesterol crystals, fibroblasts, and foreign body giant cells, confirming the diagnosis of cholesterol granuloma. The preoperative pure-tone audiometry revealed an air-bone gap in the left ear. At five months after surgery, no deterioration was observed (Figures 4A, 4B). The preoperatively identified intracranial lesion was no longer detectable on the MRI performed at the four-month postoperative follow-up (Figures 5A, 5B), suggesting spontaneous regression. At the 21-month postoperative follow-up, there was no recurrence of the intracranial lesion.

**Figure 4:**
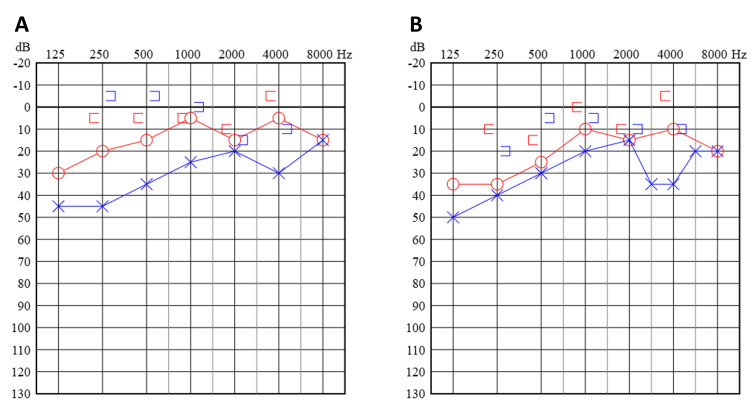
Pre- and postoperative pure-tone audiometry results (A) Preoperative audiogram. (B) Audiogram at five months postoperatively.

**Figure 5:**
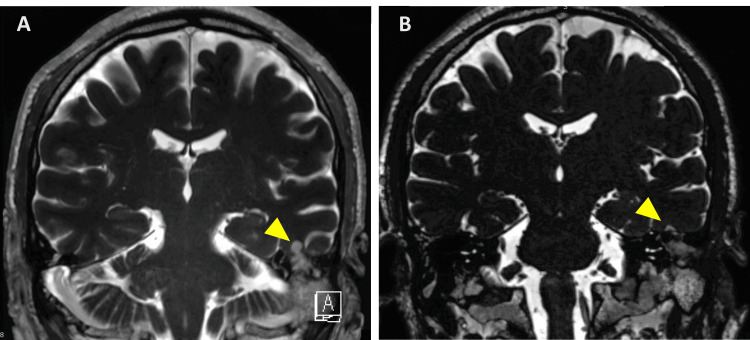
Comparison of pre- and postoperative MRI images (A) Preoperative heavily T2-weighted image (CISS). A spherical hyperintense area was observed intracranially (yellow arrowhead). (B) Postoperative heavily T2-weighted image (CISS). The intracranial lesion had disappeared (yellow arrowhead). CISS, constructive interference in steady state

## Discussion

Cholesterol granuloma is formed by a foreign body giant cell reaction to cholesterol crystals, which are breakdown products of erythrocytes, triggered by negative pressure due to ventilation failure in the middle ear cavity or hemorrhage [8,11]. In the present case, reduced ventilation of the middle ear mucosa due to prior surgery or inflammation, combined with micro-hemorrhage from the bony defect, may have contributed to granuloma formation and its subsequent intracranial extension.

A notable feature of this case is that the granuloma extending into the middle cranial fossa appeared to regress after removal of the middle ear lesion and closure of the bony defect alone. In general, for cholesterol granulomas with intracranial extension, complete resection, often involving neurosurgical approaches, has been recommended to avoid brain compression symptoms or neurological deficits associated with lesion enlargement [5-10]. However, in the present case, the intracranial lesion may have regressed through the following mechanisms, although these interpretations remain speculative because this is a single case, and no histopathological confirmation of the intracranial component was obtained.

First, given the “mushroom-like” configuration of the lesion, the communication (stalk) between the middle ear and the intracranial space appeared to be relatively narrow, suggesting that maintenance of the lesion may have depended largely on the middle ear pathology. Removal of the middle ear granulation tissue and physical closure of the bony defect with bone paté may have interrupted the supply of new hemorrhage and inflammatory exudate to the intracranial portion.

Second, resorption of the intracranial lesion may also have contributed to this outcome. Once the supply route was interrupted, the residual intracranial granuloma may have been gradually phagocytosed and absorbed via the rich blood supply of the dural surface, or it may have progressively organized and decreased in size as the inflammation subsided.

Regarding treatment strategy, in the middle ear, cholesterol granulomas are generally non-invasive [9]. Although surgical resection is the standard treatment for middle ear cholesterol granuloma with intracranial extension [5-10], this case suggests that a two-stage strategy may be effective. In cases where the intracranial lesion is small and located in a high-risk area (e.g., the dominant hemisphere) where surgical invasiveness is a concern, addressing the middle ear pathology and blocking the communication route without immediate extended surgery is a viable option. Although the patient was asymptomatic, surgical intervention was considered justified in this case. Because the patient had a history of prior surgery for cholesteatoma, recurrent cholesteatoma was initially suspected. On this basis, rather than choosing conservative follow-up alone, we opted for a surgical approach limited to the middle ear lesion. Provided that the dura is preserved and there is no suspicion of dural defects or brain parenchymal invasion intraoperatively, selecting such a minimally invasive approach with strict follow-up via MRI can be considered a valid alternative.

A major strength of this case is the demonstration that minimally invasive surgery can effectively treat intracranial extension in the dominant hemisphere without neurological sequelae. However, there are several limitations. First, this is a single case report, and the findings may not be generalizable to all intracranial cholesterol granulomas. Second, since the intracranial lesion was not resected, histopathological confirmation of the intracranial portion was not obtained. Finally, while the follow-up period of one year and nine months showed no recurrence, longer-term observation is necessary to definitively rule out late recurrence.

## Conclusions

We reported a case of middle ear cholesterol granuloma with mushroom-like extension into the middle cranial fossa, where the intracranial lesion spontaneously resolved following transmastoid removal of the middle ear lesion and closure of the bony defect. Even in cases with intracranial extension, considering the lesion characteristics and risks, improving the middle ear environment and blocking access may potentially achieve regression of the intracranial lesion.
